# Active Surveillance for Invasive Mold Disease — Four Hospitals, Atlanta, Georgia, 2020–2024

**DOI:** 10.15585/mmwr.ss7505a1

**Published:** 2026-07-30

**Authors:** Elizabeth T. Sajewski, Carolyn Mackey, Stepy Thomas, Kyle Winston, Dana Goodenough, Alison M. Binder, Andrea Cool, Donika Grandberry, Mitsuru Toda, Lalitha Gade, Anastasia P. Litvintseva, Shawn R. Lockhart, Luisa F. López, Krupali Patel, Saarang Gopinath, Bruno C. Rediguieri, Brian Min, Natalie S. Nunnally, Cordel Phillips, D. Joseph Sexton, Brian Jester, Eddie Mata, Hannah Zenas, Monica M. Farley, Stephanie M. Pouch, Lucy S. Witt, Nora T. Oliver, Jeremy A. W. Gold

**Affiliations:** ^1^Division of Foodborne, Waterborne, and Environmental Diseases, National Center for Emerging and Zoonotic Infectious Diseases, CDC, Atlanta, Georgia; ^2^Epidemic Intelligence Service, CDC, Atlanta, Georgia; ^3^Atlanta Veterans Affairs Medical Center, Decatur, Georgia; ^4^Georgia Emerging Infections Program, Atlanta, Georgia; ^5^Department of Medicine, Emory University School of Medicine, Atlanta, Georgia; ^6^Division of Healthcare Quality Promotion, National Center for Emerging and Zoonotic Infectious Diseases, CDC, Atlanta, Georgia; ^7^Booz Allen Hamilton, McLean, Virginia; ^8^GAP Solutions Inc., Herndon, Virginia; ^9^PVM, Inc., San Diego, California

## Abstract

**Problem/Condition:**

Invasive mold diseases (IMDs) are rare, life-threatening infections that primarily affect persons with immunocompromising conditions. The most common molds associated with IMDs are *Aspergillus* spp., Mucorales (e.g., *Mucor* spp., *Rhizomucor* spp., and *Rhizopus* spp.), *Fusarium* spp., and *Scedosporium* spp. IMDs can affect the lungs, sinuses, skin, central nervous system, or multiple body sites.

**Period Covered:**

2020–2024.

**Description of System:**

IMD is not a nationally notifiable condition. CDC conducts active, laboratory-based IMD surveillance through the Georgia Emerging Infections Program at three laboratories that serve four Atlanta, Georgia, hospitals: two academic hospitals and their associated outpatient clinics, one federal hospital, and one community hospital. Identifying and classifying IMD cases is challenging because 1) symptoms often are nonspecific and 2) the presence of mold growth in clinical cultures might indicate infection, colonization, or laboratory contamination.

Potential IMD cases were identified through a review of records from laboratories serving the hospitals included in this surveillance system to identify patients who received positive results from mold cultures or *Aspergillus* galactomannan tests, which detect the presence of galactomannan, a compound found in *Aspergillus *fungi, in serum or bronchoalveolar lavage fluid specimens. Potential cases in these patients were classified as proven or probable cases using established National Institute of Allergy and Infectious Diseases Mycoses Study Group (MSG) criteria that consider laboratory findings (e.g., histopathologic evidence of angioinvasive mold infection or a fungal culture from a normally sterile body site), clinical or radiologic features supporting IMD classification (e.g., chest computed tomography or bronchoscopy results suggestive of fungal disease, sinonasal signs, or central nervous system imaging results), and host factors that predispose patients to IMD (e.g., recent history of neutropenia, hematologic malignancy, transplantation receipt, and use of certain immunosuppressive medications). Potential cases that did not meet the laboratory, clinical, and host-factor criteria for MSG-proven or MSG-probable cases could be categorized as surveillance cases if treating clinicians diagnosed IMD in a patient and initiated treatment with antifungal therapy effective against mold or if the patient died within 3 days of specimen collection.

**Results:**

During 2020–2024, a total of 968 unique patients with potential IMD were identified across four Atlanta hospitals and their associated outpatient clinics. Of those, 449 (46%) were classified as having IMD, including 89 (20%) MSG-proven, 142 (32%) MSG-probable, and 218 (49%) surveillance cases. Among patients with IMD, 58 (13%) had a current or recent COVID-19 diagnosis. Information on bed count was available for three of the four hospitals. At the two academic hospitals, the pooled average annual IMD incidence was 4.8 inpatient cases per 100 inpatient beds and 14.0 intensive care unit (ICU) cases per 100 ICU beds. At the community hospital, the average annual incidence was 2.8 inpatient cases per 100 inpatient beds and 10.2 ICU cases per 100 ICU beds.

Among 449 patients with IMD, the largest percentage were aged 45–64 years (43%), followed by ≥65 years (39%), 19–44 years (18%). Patients aged 1–18 years and <1 year each accounted for <1% of IMD cases. Pulmonary infections were most common (68%), followed by cutaneous or deep tissue (nonfacial) infections (11%), sinus or nasal infections (10%), and central nervous system infections (excluding eyes) (9%); less frequent sites of infection included soft tissue (2%), blood (1%), bone (1%), and eye (1%). The most common fungal species identified among IMD cases were *Aspergillus* spp. (n = 319 [71%]), most commonly *Aspergillus fumigatus* (n = 94 [21%]). *Fusarium* spp. were the second most common (n = 20 [4%]), followed by Mucorales (n = 17 [4%]), and *Scedosporium* spp. (n = 12 [3%]). *Aspergillus* spp., *Scedosporium* spp., and *Curvularia* spp. were most commonly associated with pulmonary infections. Mucorales genera were more commonly associated with sinus, nasal, or facial infections and *Fusarium* spp. with cutaneous or deep tissue infections. Among patients with IMD, 65% (n = 292) had at least one MSG host factor that predisposes patients to IMD, and 53% (n = 238) had at least one MSG clinical criterion supporting IMD classification, suggesting that surveillance based only on classic host risk factors or typical clinical findings might miss a substantial proportion of IMD cases.

A total of 363 (81%) of 449 patients with IMD received antifungal treatment effective against molds, most commonly isavuconazole (n = 178 [40%]), followed by voriconazole (n = 142 [32%]) and amphotericin B (n = 93 [21%]). Overall, 43% of patients with IMD were admitted to an ICU during the 2 weeks preceding specimen collection, and 50% of patients with IMD required intubation and mechanical ventilation; the 90-day all-cause mortality rate, excluding 2024 cases, was 45% (157 of 349). Indicators of severe illness were more common among IMD patients with a current or recent COVID-19 diagnosis than among those without COVID-19, including higher frequency of ICU admission during the 2 weeks preceding specimen collection (66% [38 of 58] versus 39% [154 of 391]; p<0.001) and increased 90-day all-cause mortality (66% [35 of 53] versus 41% [122 of 296]; p<0.001).

**Interpretation:**

IMDs are severe infections associated with frequent ICU admission and high mortality rates, especially among patients with a current or recent COVID-19 diagnosis. The findings from this report could serve as benchmark data in establishing baseline IMD rates for future surveillance to detect outbreaks in health care settings. More than one third of IMD cases (35%) occurred in patients without MSG host factors, underscoring the need for clinicians and surveillance efforts to consider the possibility of IMDs among patients without classic host risk factors for infection.

**Public Health Action:**

Health care providers should be vigilant for IMDs as a life-threatening complication among persons with immunocompromising conditions or critical illness. Continued surveillance could help identify emerging populations at risk for IMD, facilitate earlier recognition and treatment, and support detection of health care–associated outbreaks.

## Introduction

Mold is a type of fungus that grows in multicellular filaments (hyphae). Prevalent in both outdoor and indoor environments, mold does not usually cause severe infection among patients with competent immune systems (*1*,*2*). Invasive mold diseases (IMDs) are a diverse group of rare, life-threatening infections caused by certain mold species that can invade and damage body tissues, particularly among patients with immunocompromising conditions. The most common molds associated with IMDs are *Aspergillus* spp., Mucorales (e.g., *Mucor* spp., *Rhizomucor* spp., and *Rhizopus* spp.), *Fusarium* spp., and *Scedosporium* spp. (*3*–*7*). IMDs can affect various body sites, such as the lungs, sinuses, skin, or central nervous system and can affect multiple body sites simultaneously (*8*). IMDs most often develop after the inhalation of mold spores from the environment but can also occur through traumatic inoculation by or exposure to contaminated medical devices, health care linens, or health care products (*2*,*9*–*11*). IMDs can occur sporadically through exposure to mold in community or health care settings or as part of health care–associated outbreaks (*9*,*12*,*13*). However, baseline rates of IMDs are not well-defined, making it difficult to distinguish expected sporadic cases from potential health care–associated clusters and determine when investigation for a common source is warranted. Recognized IMD risk factors include hematologic malignancy, receipt of a hematopoietic stem cell transplant or solid organ transplant, neutropenia, uncontrolled diabetes mellitus, and receipt of immunosuppressive therapy (*8*,*14*). Concurrent or recent critical illness is increasingly recognized as an IMD risk factor, particularly for patients with severe influenza and severe COVID-19 (*15*–*18*).

IMD treatment generally requires prolonged therapy with antifungal drugs effective against molds; these are limited to the triazole drugs voriconazole, itraconazole, posaconazole, and isavuconazole or the polyene amphotericin B (*19*,*20*). Echinocandins, such as caspofungin, micafungin, and anidulafungin, might also be used as part of combination therapy to enhance efficacy, particularly in severe or antifungal-resistant cases (*19*,*20*). Certain molds, particularly those from the genera *Scedosporium* and *Fusarium* or from the order Mucorales, exhibit intrinsic resistance to multiple antifungal drugs, underscoring the importance of accurate identification of the pathogen to guide clinical care. Analyses of administrative datasets estimate that in the United States, IMDs are associated with >15,000 hospitalizations and direct medical costs exceeding $1.3 billion annually (*21*). Case-fatality rates can approach 85%, depending on the causative organism, affected body site, and host characteristics (*16*,*22*,*23*).

Diagnosing and establishing surveillance for IMDs present several challenges. IMD symptoms often are nonspecific, and the presence of mold growth in clinical cultures might indicate infection, colonization, or laboratory contamination. In addition, obtaining culture or biopsy specimens for definitive diagnosis might involve invasive procedures that pose risks for severely or critically ill patients (*8*,*9*,*24*). Therefore, clinical diagnosis and treatment, as well as public health surveillance efforts, generally rely on consideration of clinical features, radiographic findings, and culture- and non-culture–based diagnostic test findings (*9*,*24*). To facilitate clinical trials for treatment of invasive fungal diseases and studies among patients at risk for these diseases, revised consensus case definitions were published in 2020 by the European Organization for Research and Treatment of Cancer/Invasive Fungal Infections Cooperative Group (EORTC) and the National Institute of Allergy and Infectious Diseases Mycoses Study Group (MSG) in the United States, which are prominent research organizations focused on the study and treatment of invasive fungal diseases (*14*). These definitions classify cases as proven, probable, or possible cases using an algorithm that considers laboratory findings, clinical factors (e.g., chest computed tomography [CT] findings), and host factors (e.g., recent history of neutropenia) (*14*).

In the United States, the public health importance of IMDs is likely to increase because of the growing population of persons living with immunosuppressive conditions, increased detection of antifungal-resistant molds, and the potential for shifts in the geographic range of pathogenic molds (*8*,*25*,*26*). Therefore, in 2017, CDC and the Georgia Emerging Infections Program (EIP) launched a pilot surveillance program conducting active, laboratory-based surveillance for IMDs at four metropolitan Atlanta hospitals and their associated outpatient clinics; the hospitals are served by three laboratories (*8*). The pilot program, which ran from 2017 through 2019, was limited to IMD in residents of an eight-county catchment area surrounding and including the Atlanta metropolitan area. In addition to ascertaining MSG-defined proven and probable IMD cases, the program also included a surveillance IMD case definition intended to identify and include clinically significant cases of IMD that did not meet MSG criteria (*8*). This definition was included to ensure that a broader spectrum of patients who might experience IMD could be represented, particularly those who might not fit the typical risk profiles or clinical presentations outlined in the MSG criteria (*8*).

Beginning in 2020, the EIP surveillance system expanded to include cases among all Georgia residents (i.e., rather than only those in the Atlanta metropolitan area) who were patients at the four Atlanta-area facilities and associated outpatient clinics and to document COVID-19 infection status among patients (*8*); in 2023, surveillance was expanded to also include cases among patients who were not Georgia residents but received care at the Atlanta-area EIP facilities. This report summarizes findings from an analysis of 2020–2024 EIP IMD surveillance data from the four Atlanta hospitals and their associated outpatient clinics. Health care providers, public health officials, and clinical researchers can use these findings to help increase awareness of IMDs and to guide prevention, diagnostic, treatment, and surveillance practices.

## Methods

### Data Sources

IMD is not a nationally notifiable condition. CDC conducts active, laboratory-based IMD surveillance through the Georgia EIP at three laboratories, which serve two academic hospitals and their associated outpatient clinics, one federal hospital, and one community hospital. Georgia EIP is part of a network of 12 U.S. sites that, in collaboration with CDC, works with health care systems, academic institutions, and state and local public health departments to conduct active surveillance for emerging infectious diseases.

### Ascertainment of Potential Cases

**FIGURE 1:**
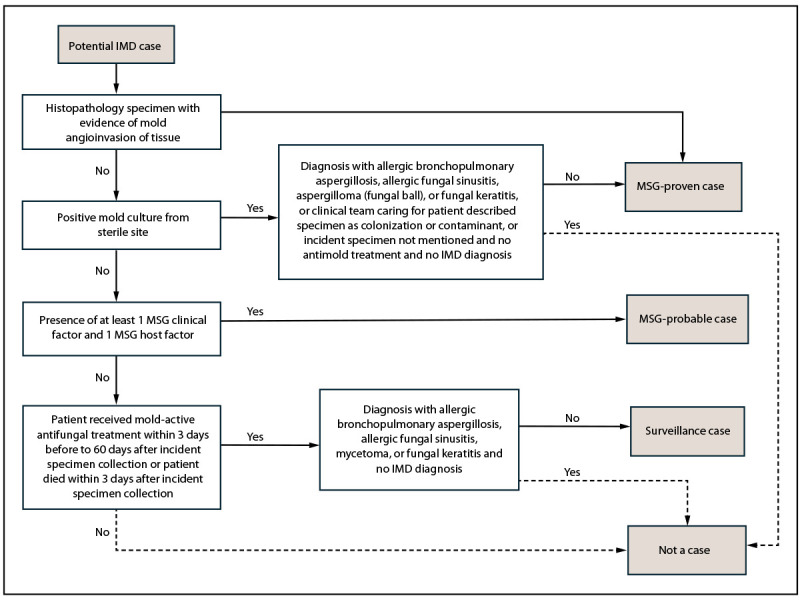
Criteria for identifying proven, probable, and surveillance cases of invasive mold disease*^,†^ — four hospitals, Atlanta, Georgia, 2020–2024 **Abbreviations:** IMD = invasive mold disease; MSG = National Institute of Allergy and Infectious Diseases Mycoses Study Group criteria. * IMD cases meet a combination of criteria, including mycologic evidence (e.g., culture or histopathologic findings), clinical factors (e.g., chest computed tomography findings), host factors (e.g., recent history of neutropenia or ever having received a solid organ transplant), and clinician suspicion for IMD as indicated by patient receipt of mold-active antifungal treatment. Potential cases determined to not be cases are included in the surveillance system on the basis of a positive mold culture or a positive *Aspergillus* galactomannan test (serum or bronchoalveolar lavage specimen with index value ≥0.5) but do not meet criteria for an IMD case. ^†^ Potential cases were classified as MSG-proven or MSG-probable cases based on established criteria (https://doi.org/10.1093/cid/ciz1008). Surveillance cases were classified as potential cases if they did not meet the criteria for MSG-proven or MSG-probable cases for which the clinician initiated treatment with antifungal therapy effective against mold or if the patient died within 3 days of specimen collection. For potential cases meeting MSG-proven and surveillance case criteria, if factors indicated a non-IMD (e.g., non-IMD diagnoses or clinical team described specimens as having colonization or contaminant) and no IMD diagnosis was indicated, the potential cases were determined to not be IMD cases.

Potential IMD cases were identified through a review of records from clinical, reference, and commercial laboratories serving the hospitals included in this surveillance system to identify patients who received a positive mold culture or *Aspergillus* galactomannan test result (Figure 1). Positive mold culture specimens included in the surveillance system were required to meet body site–specific and species-specific criteria intended to exclude specimens that likely represent colonization or environmental contamination (e.g., *Penicillium* spp. in a sputum specimen) or noninvasive mold disease (e.g., mold isolated from hair or nails). Because mold colonization is particularly common in lung specimens from patients with cystic fibrosis (*27*), case classifications and full chart abstractions for specimens collected from patients with cystic fibrosis were not performed; these specimens were archived for potential future study. A full list of exclusion criteria for culture specimens is available (Supplementary Box). The *Aspergillus* galactomannan test is a diagnostic tool used to help diagnose invasive aspergillosis; the test detects the presence of galactomannan, a compound found in *Aspergillus* fungi, in serum or bronchoalveolar lavage fluid specimens. A positive *Aspergillus* galactomannan test result was defined as a serum or bronchoalveolar lavage specimen with an index value ≥0.5; this index value is a semiquantitative measure of galactomannan antigen levels calculated by comparing the optical density of the patient’s specimen with that of a control specimen. This index value cutoff was chosen, rather than the EORTC and MSG cutoff of 1.0 (proposed in 2020), to increase the sensitivity of the surveillance system for ascertaining potential cases (*8*,*14*).

In addition to mold culture findings and *Aspergillus* galactomannan testing, the 2017–2019 pilot IMD surveillance program included screening of unstructured text from histopathology reports to ascertain potential IMD cases. However, beginning in 2020, this process was discontinued because of time constraints and limited yield: among 1,698 histopathology reports reviewed during the pilot study period, only seven IMD cases (0.4%) were identified that would not have been identified by review of *Aspergillus* galactomannan testing or mold culture results (*8*). Although histopathology findings were not used to ascertain potential cases, information was extracted from histopathology reports associated with potential cases identified through culture results or *Aspergillus* galactomannan testing to support case classification and provide additional information on potential cases included in the surveillance system.

### IMD Case Definitions

On the basis of certain microscopic, cytopathologic, or other laboratory findings, potential IMD cases were classified as either cases (MSG-proven cases, MSG-probable cases, or surveillance cases) or not cases. A proven MSG case met at least one of the following criteria: 1) detection of hyphae or melanized yeast-like forms, along with evidence of associated tissue damage, using histopathologic, cytopathologic, or direct microscopic examination of a specimen obtained through needle aspiration or biopsy; 2) recovery of hyaline or pigmented mold from a specimen obtained by a sterile procedure from a normally sterile and clinically or radiologically abnormal site, excluding bronchoalveolar lavage fluid, paranasal or mastoid sinus cavity specimens, and urine; or 3) detection of mold on a blood specimen culture for a patient with signs and symptoms consistent with IMD (*14*).

MSG-probable cases did not meet MSG-proven case criteria but had at least one host factor that predisposes patients to IMD and one clinical or radiologic factor supporting IMD classification. Host factors predisposing patients to IMD included a history of neutropenia (<500 neutrophils/mm^3^) for >10 of the previous 30 days, the presence of a hematologic malignancy in the previous 2 years, receipt of an allogeneic stem cell or solid organ transplant at any time, receipt of >21 days of systemic corticosteroids at a dose equivalent to ≥20 mg prednisone during the previous 60 days, receipt of noncorticosteroid immunosuppressive medications during the previous 90 days, inherited immunodeficiency or severe autoimmune disease during the previous 2 years, or acute graft-versus-host disease grade III or IV during the previous 90 days. Clinical or radiologic factors supporting IMD classification included patterns observed on CT findings consistent with pulmonary aspergillosis (e.g., dense well-circumscribed lesions or wedge-shaped consolidations), certain bronchoscopy findings (e.g., presence of ulcer, nodule, or eschar), signs of sinonasal disease (e.g., nasal ulcer with black eschar or visual signs and symptoms), or central nervous system infection (e.g., head CT with a lesion or meningeal enhancement) (*14*).

Potential cases that did not meet the criteria for MSG-proven or MSG-probable cases, such as IMD occurring in a previously healthy person who developed severe COVID-19, could be categorized as surveillance cases. A surveillance case was defined as one in which the treating clinicians diagnosed IMD in a patient and initiated treatment with antifungal therapy effective against mold; however, the patient’s illness did not meet the full criteria for an MSG-proven or MSG-probable case. The use of antifungal prophylaxis to prevent fungal infections did not qualify as treatment in this context. In addition, the potential cases in patients who died within 3 days of the incident mold specimen collection date were classified as surveillance cases, regardless of whether they received antifungal therapy. This inclusion accounted for IMD in patients who might have died before a clinician had the opportunity to initiate antifungal treatment.

Patients with potential IMD that did not meet MSG-proven, MSG-probable, or surveillance case criteria and did not die within 3 days of specimen collection were not classified as having IMD. In addition, regardless of whether MSG or surveillance criteria were met, potential cases could be classified as not being IMD cases if the treating clinician indicated suspicion that a positive mold specimen was the result of laboratory contamination or colonization or the clinician did not address the mold specimen in clinical notes or administer antifungal therapy for IMD. Finally, a potential case was not classified as an IMD case if the treating clinician’s primary diagnosis was one of the following noninvasive mold diseases: allergic bronchopulmonary aspergillosis, allergic fungal sinusitis, aspergilloma (fungal ball), or fungal keratitis. If a patient received a diagnosis of a noninvasive mold infection and an IMD diagnosis (e.g., the patient had both allergic bronchopulmonary aspergillosis and invasive pulmonary aspergillosis), the case was classified as IMD.

### Laboratory Methods

Speciation of isolates from mold culture specimens associated with potential IMD cases was conducted by the clinical, reference, or commercial laboratories associated with each health care facility and according to current recommended laboratory practices. Georgia EIP obtained and sent isolates (up to four specimens per potential case) to CDC for species confirmation at CDC’s Mycotic Diseases Branch reference laboratory. Isolates were subcultured on Sabouraud dextrose agar plates and incubated at 30°C (86°F) for 2–12 days, until sufficient growth was observed. DNA extraction was performed using the Qiagen DNeasy Blood and Tissue Kit following the manufacturer’s instructions. Targeted polymerase chain reaction amplification and DNA sequencing were performed for species confirmation (*28*–*30*). Genetic data were analyzed using Geneious Prime software (version 2022.1.1; Dotmatics) and then compared with known sequences in the National Center for Biotechnology Information (NCBI) GenBank using the Basic Local Alignment Search Tool (BLAST) algorithm (*28*). CDC laboratory findings were not used to guide clinical care.

### Data Analysis

Surveillance data were analyzed at the patient level. One case was considered per patient on the basis of the collection date of each patient's first positive mold culture or *Aspergillus* galactomannan test result during the surveillance period. Surveillance data were matched with Georgia Department of Public Health Vital Statistics registry data to identify patients who died within 90 days of the incident mold specimen collection for calculation of 90-day all-cause mortality. In addition, surveillance data were matched with information from Georgia SARS-CoV-2 infection registries to identify patients with SARS-CoV-2 infection; this process aimed to validate and enhance SARS-CoV-2 information obtained from medical chart review. IMD cases with COVID-19 were defined as those in patients with IMD who also had received a positive SARS-CoV-2 test result documented from 90 days before through the date of incident mold specimen collection. To calculate the average annual incidence of IMD among all inpatients and among intensive care unit (ICU) patients, the average annual numbers of staffed inpatient and ICU beds per facility were used, as reported to the National Healthcare Safety Network (NHSN) during August 1, 2020–December 31, 2022.

Potential cases were tabulated and examined quarterly, stratifying by IMD case versus not a case status and, among IMD cases, by MSG case type (i.e., MSG-proven, MSG-probable, and surveillance case) and by recent or current COVID-19 status. Demographic features, clinical characteristics, health care utilization, outcomes, and diagnostic testing were examined. For these comparisons, p-values (α = 0.05) were calculated using the Fisher’s exact test for categorical variables and the Kruskal–Wallis H test for continuous variables. Mold species were examined by case type and affected body site. Analyses were conducted using R software (version 4.4.0; R Foundation). This activity was reviewed by CDC, deemed not research, and conducted consistent with applicable federal law and CDC policy.* The activity also was deemed not research by the Georgia Department of Public Health and Emory University institutional review boards.

## Results

### Case Ascertainment, Case Classification, and Temporal Trends

**FIGURE 2:**
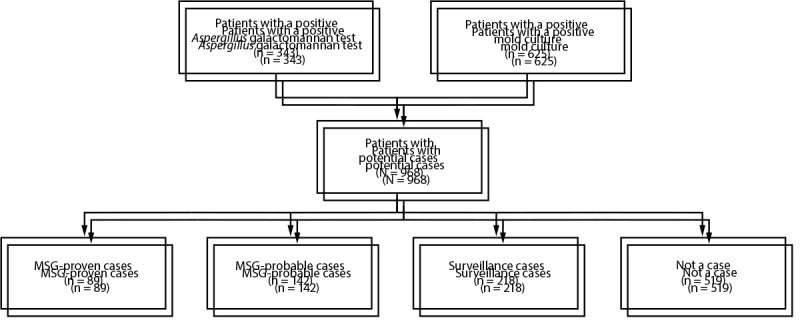
Invasive mold disease case classification*^,†^ — four hospitals, Atlanta, Georgia, 2020–2024 **Abbreviation:** MSG = National Institute of Allergy and Infectious Diseases Mycoses Study Group criteria. * IMD cases meet a combination of criteria, including mycologic evidence (e.g., culture or histopathologic findings), clinical factors (e.g., chest computed tomography findings), host factors (e.g., recent history of neutropenia or ever having received a solid organ transplant), and clinician suspicion for IMD as indicated by patient receipt of mold-active antifungal treatment. Potential cases determined to not be cases are included in the surveillance system on the basis of a positive mold culture or a positive *Aspergillus* galactomannan test (serum or bronchoalveolar lavage specimen with index value ≥0.5) but do not meet criteria for an IMD case. ^†^ Potential cases were classified as MSG-proven or MSG-probable cases based on established criteria (https://doi.org/10.1093/cid/ciz1008). Surveillance cases were classified as potential cases if they did not meet the criteria for MSG-proven or MSG-probable cases for which the clinician initiated treatment with antifungal therapy effective against mold or if the patient died within 3 days of specimen collection. For potential cases meeting MSG-proven and surveillance case criteria, if factors indicated a non-IMD (e.g., non-IMD diagnoses or clinical team described specimens as having colonization or contaminant) and no IMD diagnosis was indicated, the potential cases were determined to not be IMD cases.

**FIGURE 3:**
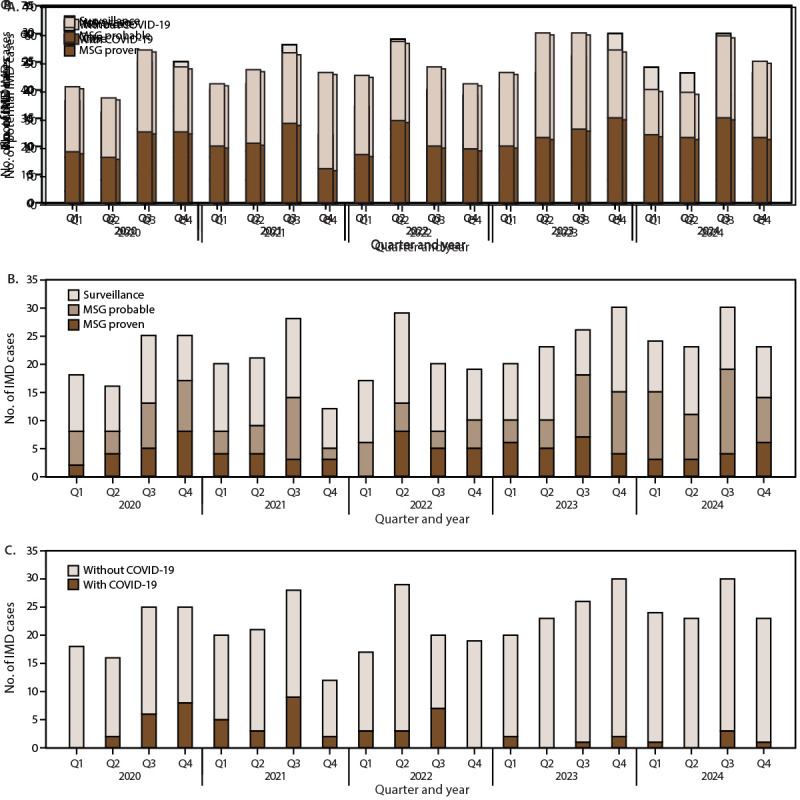
Cases of invasive mold disease, by case status (A),* invasive mold disease case definition (B),^†^ and COVID-19 status (C) — four hospitals, Atlanta, Georgia, 2020–2024^§^ **Abbreviations:** IMD = invasive mold disease; MSG = National Institute of Allergy and Infectious Diseases Mycoses Study Group criteria. * IMD cases meet a combination of criteria, including mycologic evidence (e.g., culture or histopathologic findings), clinical factors (e.g., chest computed tomography findings), host factors (e.g., recent history of neutropenia or ever having received a solid organ transplant), and clinician suspicion for IMD as indicated by patient receipt of mold-active antifungal treatment. Potential cases determined to not be cases are included in the surveillance system on the basis of a positive mold culture or a positive *Aspergillus* galactomannan test (serum or bronchoalveolar lavage specimen with index value ≥0.5) but do not meet criteria for an IMD case. ^†^ Potential cases were classified as MSG-proven or MSG-probable cases based on established criteria (https://doi.org/10.1093/cid/ciz1008). Surveillance cases were classified as potential cases if they did not meet the criteria for MSG-proven or MSG-probable cases for which the clinician initiated treatment with antifungal therapy effective against mold or if the patient died within 3 days of specimen collection. For potential cases meeting MSG-proven and surveillance case criteria, if factors indicated a non-IMD (e.g., non-IMD diagnoses or clinical team described specimens as having colonization or contaminant) and no IMD diagnosis was indicated, the potential cases were determined to not be IMD cases. ^§^ Q1 = January–March; Q2 = April–June; Q3 = July–September; Q4 = October–December.

Surveillance staff members reviewed positive specimens collected during 2020–2024 by participating facilities and, after the application of exclusion criteria, identified 968 unique patients with potential IMD (Figure 2). Among the excluded cases were those in 96 patients with cystic fibrosis and 22 patients during 2020–2022 who were out-of-state residents. Index specimens for potential cases included 343 (35%) specimens with positive *Aspergillus* galactomannan test results and 625 (65%) with positive mold cultures. Among the 968 potential cases, 449 (46%) were classified as IMD cases, including 89 MSG-proven (20%), 142 MSG-probable (32%), and 218 surveillance cases (49%) (Figure 3). Among all IMD cases, 58 (13%) were identified in patients with a recent or current COVID-19 diagnosis.

During 2020–2024, the total annual number of potential cases ranged from 180 to 220, with 81–100 IMD cases identified per year. The relative proportion of patients with IMD versus those without IMD and the proportional distribution by case type were generally stable, suggesting no substantial changes in IMD epidemiology within the surveillance population over the study period (Figure 3). The proportion of IMD cases with COVID-19 peaked at 23% (n = 19 of 81) in 2021 and was lowest (5%) in 2024 (n = 5 of 100). 

Most potential cases (n = 756 of 968 [78%]) and most IMD cases (n = 360 of 449 [80%]) were identified from the two academic hospitals or their associated outpatient clinics, followed by the community hospital (n = 70 of 449 [16%]); only 4% (19 of 449) were identified from the federal hospital (Table 1). Overall, 81% (362 of 449) of IMD cases were identified in inpatients, a substantially higher percentage than the 44% (n = 230 of 519) among inpatients with potential cases determined not to be IMD cases. Bed capacities were available from NHSN for three of the four hospitals included in the surveillance system, including two hospitals within the same academic hospital system and the community hospital. Bed capacity values were used to calculate annual IMD incidence by hospital. At the two academic hospitals, the pooled average annual IMD incidence was 4.8 inpatient cases per 100 inpatient beds and 14.0 ICU cases per 100 ICU beds. At the community hospital, the average annual IMD incidence was 2.8 inpatient cases per 100 inpatient beds and 10.2 ICU cases per 100 ICU beds.

**TABLE 1:** Demographic characteristics of patients with invasive mold disease — four hospitals, Atlanta, Georgia, 2020–2024*

Characteristic	Potential IMD cases^†^			IMD cases by case type				IMD cases by COVID-19 status^§^		
	IMD case (n = 449)	Not an IMD case (n = 519)	p-value	MSG proven (n = 89)	MSG probable (n = 142)	Surveillance (n = 218)	p-value	With COVID-19 (n = 58)	Without COVID-19 (n = 391)	p-value
	No. (%)	No. (%)		No. (%)	No. (%)	No. (%)		No. (%)	No. (%)	
**Year of incident mold specimen collection**										
2020	84 (19)	96 (18)	0.30	19 (21)	27 (19)	38 (17)	0.16	16 (28)	68 (17)	<0.001
2021	81 (18)	107 (21)		14 (16)	22 (15)	45 (21)		19 (33)	62 (16)	
2022	85 (19)	107 (21)		18 (20)	19 (13)	48 (22)		13 (22)	72 (18)	
2023	99 (22)	121 (23)		22 (25)	31 (22)	46 (21)		5 (9)	94 (24)	
2024	100 (22)	88 (17)		16 (18)	43 (30)	41 (19)		5 (9)	95 (24)	
**Median age, yrs (IQR)**	60 (50–69)	63 (52–72)	0.046	58 (50–69)	60 (52–68)	62 (47–69)	0.70	64 (53–72)	60 (49–68)	0.04
**Age group, yrs**										
<1	1 (<1)	1 (<1)	0.01	0 (—)	0 (—)	1 (<1)	0.003	0 (—)	1 (<1)	0.47
1–18	1 (<1)	7 (1)		0 (—)	1 (1)	0 (—)		0 (—)	1 (<1)	
19–44	82 (18)	77 (15)		16 (18)	16 (11)	50 (23)		9 (16)	73 (19)	
45–64	192 (43)	191 (37)		44 (49)	74 (52)	74 (34)		21 (36)	171 (44)	
≥65	173 (39)	243 (47)		29 (33)	51 (36)	93 (43)		28 (48)	145 (37)	
**Sex**										
Female	159 (35)	229 (44)	0.007	27 (30)	59 (42)	73 (33)	0.16	22 (38)	137 (35)	0.66
Male	290 (65)	290 (56)		62 (70)	83 (58)	145 (67)		36 (62)	254 (65)	
Race and ethnicity^¶,^**										
Asian	21 (5)	7 (1)	0.03	4 (4)	3 (2)	14 (6)	0.17	2 (3)	19 (5)	0.55
Black or African American	175 (39)	213 (41)		33 (37)	63 (44)	79 (36)		24 (41)	151 (39)	
White	208 (46)	255 (49)		41 (46)	68 (48)	99 (45)		27 (47)	181 (46)	
Other races**	1 (<1)	0 (—)		0 (—)	0 (—)	1 (<1)		0 (—)	1 (<1)	
Hispanic or Latino	27 (6)	23 (4)		5 (6)	7 (5)	15 (7)		5 (9)	22 (6)	
Unknown	17 (4)	21 (4)		6 (7)	1 (1)	10 (5)		0 (—)	17 (4)	
**Facility type of incident mold specimen collection**										
Academic hospital	360 (80)	396 (76)	0.008	72 (81)	126 (89)	162 (74)	<0.001	48 (83)	312 (80)	0.31
Federal hospital	19 (4)	48 (9)		3 (3)	10 (7)	6 (3)		4 (7)	15 (4)	
Community hospital	70 (16)	75 (14)		14 (16)	6 (4)	50 (23)		6 (10)	64 (16)	
**Location of patient on DISC**										
Inpatient	362 (81)	230 (44)	<0.001	74 (84)	118 (83)	170 (78)	0.17	53 (91)	309 (79)	0.06
Outpatient	85 (19)	288 (55)		13 (15)	24 (17)	48 (22)		5 (9)	80 (20)	
Other^††^	2 (<1)	1 (<1)		2 (2)	0 (—)	0 (—)		0 (—)	2 (<1)	
**Patient admitted to the hospital ≥7 days before DISC**	170 (38)	79 (15)	<0.001	34 (38)	47 (33)	89 (41)	0.34	32 (55)	138 (35)	0.005
**Patient location before admission, or, if not hospitalized, 3 days before DISC**										
Private residence	407 (91)	490 (94)	0.01	82 (92)	132 (93)	193 (89)	0.84	48 (83)	359 (92)	0.02
Acute care hospital	25 (6)	13 (3)		6 (7)	7 (5)	12 (6)		5 (9)	20 (5)	
Long-term care facility or long-term acute care hospital	10 (2)	4 (1)		1 (1)	2 (1)	7 (3)		5 (9)	5 (1)	
Unhoused	5 (1)	11 (2)		0 (—)	1 (1)	4 (2)		0 (—)	5 (1)	
Other^§§^	2 (<1)	1 (<1)		0 (—)	0 (—)	2 (1)		0 (—)	2 (1)	
**MSG case type^¶¶^**										
Proven	89 (20)	0 (—)	<0.001	89 (100)	0 (—)	0 (—)	<0.001	5 (9)	84 (21)	0.01
Probable	142 (32)	0 (—)		0 (—)	142 (100)	0 (—)		15 (26)	127 (32)	
Surveillance	218 (49)	0 (—)		0 (—)	0 (—)	218 (100)		38 (66)	180 (46)	

**Abbreviations:** DISC = date of incident mold specimen collection; IMD = invasive mold disease; MSG = National Institute of Allergy and Infectious Diseases Mycoses Study Group criteria.

* Data are shown as no. (column %) unless otherwise indicated. p-values were calculated using Fisher’s exact test for categorical variables and the Kruskal–Wallis H test for continuous variables.

^†^ IMD cases meet a combination of criteria including mycologic evidence (e.g., culture or histopathologic findings), clinical factors (e.g., chest computed tomography findings), host factors (e.g., recent history of neutropenia or ever having received a solid organ transplant), and clinician suspicion for IMD as indicated by patient receipt of mold-active antifungal treatment. Potential cases determined not to be IMD cases are included in the surveillance system on the basis of a positive mold culture or a positive *Aspergillus* galactomannan test (serum or bronchoalveolar lavage specimen with index value ≥0.5) but do not meet criteria for an IMD case.

^§^ IMD cases with COVID-19 were defined as IMD in patients who also had received a positive SARS-CoV-2 test result documented from 90 days before through DISC.

^¶^ Persons of Hispanic or Latino (Hispanic) origin might be of any race but are categorized as Hispanic; all racial groups are non-Hispanic.

** Includes one American Indian or Alaska Native patient; no patients identified as Native Hawaiian or other Pacific Islander or multiracial.

^††^ Other locations of patients at time of specimen collection included an outreach laboratory (one patient without IMD), an operating room (one patient with MSG-proven IMD), and an unknown location (one patient with MSG-proven IMD).

^§§^ Other locations of patients before DISC included on vacation outside the United States (not a private Georgia residence; one patient within the surveillance group), in utero (one neonatal patient within the surveillance group), and an unknown location (one patient without IMD).

**^¶¶^** IMD cases were categorized as MSG-proven, MSG-probable, or surveillance cases on the basis of criteria established by the European Organization for Research and Treatment of Cancer/Invasive Fungal Infections Cooperative Group and MSG consensus definitions for invasive fungal diseases, as revised in 2020 (https://doi.org/10.1093/cid/ciz1008). An MSG-proven case meets laboratory testing criteria of either 1) a positive mold culture from a sterile site or 2) histopathologic evidence of mold angioinvasion of tissue. An MSG-probable case meets clinical and host criteria with at least one clinical factor (e.g., chest computed tomography findings) and one host factor (e.g., recent neutropenia or receipt of a solid organ transplant). Surveillance cases do not meet MSG-proven or MSG-probable criteria but have clinical suspicion for IMD, as indicated by receipt of mold-active antifungal treatment or death within 3 days of specimen collection, potentially precluding receipt of treatment.

At the community hospital, a higher percentage of cases were classified as surveillance cases (n = 50 of 70 [71%]) compared with the academic hospitals (n = 162 of 360 [45%]) and federal hospital (n = 6 of 19 [32%]) (Table 1). At the federal hospital, MSG-probable cases (n = 10 of 19 [53%]) represented the most frequent case type, compared with 35% (126 of 360) at the academic hospitals and 9% (6 of 70) at the community hospital. No substantial differences were observed in the proportion of IMD cases with COVID-19 by facility type.

### Demographic Characteristics

Overall, minor differences were observed in patient age, sex, and race and ethnicity for patients with IMD compared with those without IMD (Table 1). Among patients with IMD (n = 449), the largest percentage were aged 45–64 years (43%), followed by those aged ≥65 years (39%), 19–44 years (18%), 1–18 years (<1%), and <1 year (<1%). Approximately two thirds (65%) of patients with IMD were male. The largest proportion of patients with IMD were White (46%), followed by Black or African American (39%), Hispanic or Latino (6%), Asian (5%), and unknown race or ethnicity (4%). Persons of Hispanic or Latino ethnicity could be of any race but were categorized as Hispanic; all racial groups were non-Hispanic. Patients determined to not have IMD were older (47% aged ≥65 years), less often male (56%), and less often Asian (1%) than those with IMD. The incident mold specimen was more frequently collected in the inpatient setting for patients with IMD compared with patients without IMD (n = 362 of 449 [81%] versus n = 230 of 519 [44%], respectively; p<0.001). Patient sex, race and ethnicity, and setting of incident mold specimen collection were similar for IMD cases with COVID-19 and IMD cases without COVID-19 and across MSG-proven, MSG-probable, and surveillance cases. Age group distribution was similar among IMD cases with and without COVID-19 but differed across case types. 

### Clinical Characteristics

Body sites of infection or colonization were generally similar among patients with and without IMD; however, patients with IMD were significantly more likely to have more than one body site identified (9% [n = 40 of 449]) than were those without IMD (3% [n = 15 of 519]) (p<0.001) (Table 2). Among all 449 IMD cases, pulmonary infections were the most common (68%), followed by cutaneous or deep tissue (nonfacial) (11%), sinus or nasal (10%), and central nervous system (excluding eyes) (9%); less frequent sites included soft tissue (2%), blood (1%), bone (1%), and eye (1%). A higher proportion of MSG-proven cases had sinus, nasal, or facial infections (27%) compared with MSG-probable (10%) and surveillance (4%) cases (p<0.001). MSG-probable cases were more frequently associated with pulmonary infections (93%) compared with MSG-proven (22%) and surveillance (70%) cases (p<0.001). Infection site did not differ significantly between IMD cases with and without a current or recent COVID-19 diagnosis; however, IMD cases with infection site not specified were more common among patients with COVID-19.

**TABLE 2:** Site of infection and diagnostic testing among patients with invasive mold disease — four hospitals, Atlanta, Georgia, 2020–2024*

Characteristic	Potential IMD cases^†^			IMD cases by case type				IMD cases by COVID-19 status^§^		
	IMD case (n = 449)	Not an IMD case (n = 519)	p-value	MSG proven (n = 89)	MSG probable (n = 142)	Surveillance (n = 218)	p-value	With COVID-19 (n = 58)	Without COVID-19 (n = 391)	p-value
	No. (%)	No. (%)		No. (%)	No. (%)	No. (%)		No. (%)	No. (%)	
**Site of infection or colonization^¶^**										
Pulmonary system	304 (68)	351 (68)	1	20 (22)	132 (93)	152 (70)	<0.001	42 (72)	262 (67)	0.46
Sinus, nasal, or facial	47 (10)	50 (10)	0.67	24 (27)	14 (10)	9 (4)	<0.001	5 (9)	42 (11)	0.82
Cutaneous or deep tissue (nonfacial)	48 (11)	49 (9)	0.52	11 (12)	0 (—)	37 (17)	<0.001	3 (5)	45 (12)	0.18
Central nervous system (excluding eyes)	41 (9)	36 (7)	0.23	19 (21)	17 (12)	5 (2)	<0.001	3 (5)	38 (10)	0.34
Eye	4 (1)	0 (—)	0.046	1 (1)	0 (—)	3 (1)	0.44	0 (—)	4 (1)	1
Other site**	37 (8)	34 (7)	0.33	32 (36)	0 (—)	5 (2)	<0.001	3 (5)	34 (9)	0.45
More than one site^††^	40 (9)	15 (3)	<0.001	16 (18)	20 (14)	4 (2)	<0.001	3 (5)	37 (9)	0.46
Site not specified (positive serum *Aspergillus* galactomannan test result only; no other indicators of infectious site)	11 (2)	14 (3)	0.84	0 (—)	0 (—)	11 (5)	0.001	5 (9)	6 (2)	0.008
**Specimen type^§§^**										
Culture positive for mold only	227 (51)	349 (67)	<0.001	73 (82)	27 (19)	127 (58)	<0.001	25 (43)	202 (52)	0.43
*Aspergillus* galactomannan antigen (serum or bronchoalveolar lavage with index value ≥0.5) only	164 (37)	162 (31)		7 (8)	98 (69)	59 (27)		24 (41)	140 (36)	
Both culture and *Aspergillus* galactomannan antigen positive	58 (13)	8 (2)		9 (10)	17 (12)	32 (15)		9 (16)	49 (13)	
MSG-proven criteria met^¶¶,^***	89 (20)	22 (4)	<0.001	89 (100)	0 (—)	0 (—)	<0.001	5 (9)	84 (21)	0.02
Histopathology consistent with IMD^†††^	61 (14)	2 (<1)	<0.001	61 (69)	0 (—)	0 (—)	<0.001	2 (3)	59 (15)	0.01
Culture from a sterile site consistent with an infectious disease process	40 (9)	20 (4)	0.001	40 (45)	0 (—)	0 (—)	<0.001	3 (5)	37 (9)	0.46

**Abbreviations:** IMD = invasive mold disease; MSG = National Institute of Allergy and Infectious Diseases Mycoses Study Group criteria.

* Data are shown as no. (column %). p-values were calculated using Fisher’s exact test.

^†^ IMD cases meet a combination of criteria including mycologic evidence (e.g., culture or histopathologic findings), clinical factors (e.g., chest computed tomography findings), host factors (e.g., recent history of neutropenia or ever having received a solid organ transplant), and clinician suspicion for IMD as indicated by patient receipt of mold-active antifungal treatment. Potential cases determined not to be IMD cases are included in the surveillance system on the basis of a positive mold culture or a positive *Aspergillus* galactomannan test (serum or bronchoalveolar lavage specimen with index value ≥0.5) but do not meet criteria for an IMD case.

^§^ IMD cases with COVID-19 were defined as IMD in patients who also had received a positive SARS-CoV-2 test result documented from 90 days before through DISC.

^¶^ Individual sites of infection or colonization listed are not mutually exclusive. Patients with multiple sites of colonization or infection are counted for both each individual site of infection as well as in the multiple infection site count.

** Other sites of infection among patients with IMD most commonly reported included soft tissue (seven patients), blood (six) and bone (five). Among patients without IMD, other sites of colonization most commonly reported included soft tissue (16 patients), internal body site (eight), and urine (four).

^††^ Among IMD cases with multiple sites of infection, the most common combination of sites reported was pulmonary and central nervous system.

^§§^ Mold cultures from noninvasive specimen sites (e.g., hair and nails), specific specimen site and species combinations, patients with cystic fibrosis (in whom mold colonization is common), and nonmold fungal species are excluded.

^¶¶^ IMD cases were categorized as MSG-proven, MSG-probable, or surveillance cases on the basis of criteria established by the European Organization for Research and Treatment of Cancer/Invasive Fungal Infections Cooperative Group and MSG consensus definitions for invasive fungal diseases, as revised in 2020 (https://doi.org/10.1093/cid/ciz1008). An MSG-proven case meets laboratory testing criteria of either 1) a positive mold culture from a sterile site or 2) histopathologic evidence of mold angioinvasion of tissue. An MSG-probable case meets clinical and host criteria with at least one clinical factor (e.g., chest computed tomography findings) and one host factor (e.g., recent neutropenia or receipt of a solid organ transplant). Surveillance cases do not meet MSG-proven or MSG-probable criteria but have clinical suspicion for IMD, as indicated by receipt of mold-active antifungal treatment or death within 3 days of specimen collection, potentially precluding receipt of treatment.

*** Certain potential IMD cases met MSG-proven case criteria but had non-IMD designations, including allergic fungal sinusitis (one case); fungal ball, aspergilloma, or mycetoma (one); suspected colonization or laboratory contamination (seven); fungal keratitis (two); or no antifungal treatment or documentation of infection in the medical record (11). These potential cases were determined not to be IMD cases.

^†††^ Histopathologic specimen in which hyphae are visualized along with evidence of mold angioinvasion of tissue as detailed in the European Organization for Research and Treatment of Cancer/Invasive Fungal Infections Cooperative Group and MSG consensus definitions for invasive fungal disease, as revised in 2020 (https://doi.org/10.1093/cid/ciz1008).

The most common fungal taxa identified among IMD cases were *Aspergillus* spp. (n = 319 [71%]), primarily *Aspergillus fumigatus* (n = 94 [21%]) (Table 3). *Fusarium* was the second most common genus (n = 20 [4%]), followed by Mucorales genera (n = 17 [4%]), and *Scedosporium* (n = 12 [3%]). *Aspergillus* spp., *Scedosporium* spp., and *Curvularia* spp. were most commonly associated with pulmonary infections (Supplementary Table). Mucorales genera were more commonly associated with sinus, nasal, or facial infections and *Fusarium* spp. with cutaneous or deep tissue infections. Among IMD cases with COVID-19, *Aspergillus* remained the most common genus identified (n = 48 of 58 [83%]). Similar proportions of IMD cases were caused by Mucorales genera among those with COVID-19 (n = 3 of 58 [5%]) compared with those without COVID-19 (n = 14 of 391 [4%]).

**TABLE 3:** Mold taxa identified among patients with invasive mold disease — four hospitals, Atlanta, Georgia, 2020–2024

Mold taxon identified	Total patients (N = 968)	Without IMD (n = 519)	IMD cases by case type*^,†^			IMD cases by COVID-19 status^§^		
			MSG proven (n = 89)	MSG probable (n = 142)	Surveillance (n = 218)	With COVID-19 (n = 58)	Without COVID-19 (n = 391)	Total cases (n = 449)
	No. (%)	No. (%)	No. (%)	No. (%)	No. (%)	No. (%)	No. (%)	No. (%)
*Aspergillus* spp.	**669 (69)**	350 (67)	41 (46)	126 (89)	152 (70)	48 (83)	271 (69)	**319 (71)**
*Aspergillus fumigatus*	**175 (18)**	81 (16)	23 (26)	18 (13)	53 (24)	18 (31)	76 (19)	**94 (21)**
*Aspergillus* spp. (non-*fumigatus*)^¶^	**122 (13)**	80 (15)	9 (10)	4 (3)	29 (13)	3 (5)	39 (10)	**42 (9)**
*Aspergillus* (species not specified)**	**368 (38)**	188 (36)	9 (10)	103 (73)	68 (31)	27 (47)	153 (39)	**180 (40)**
More than one *Aspergillus* species	**4 (<1)**	1 (<1)	0 (—)	1 (1)	2 (1)	0 (—)	3 (1)	**3 (1)**
*Fusarium* spp.	**36 (4)**	16 (3)	7 (8)	2 (1)	11 (5)	0 (—)	20 (5)	**20 (4)**
Mucorales (e.g., *Rhizopus* and *Mucor *spp.)	**27 (3)**	10 (2)	10 (11)	0 (—)	7 (3)	3 (5)	14 (4)	**17 (4)**
*Scedosporium* spp.	**25 (3)**	13 (3)	4 (4)	1 (1)	7 (3)	0 (—)	12 (3)	**12 (3)**
*Curvularia* spp.	**25 (3)**	16 (3)	3 (3)	2 (1)	4 (2)	1 (2)	8 (2)	**9 (2)**
*Penicillium* spp.	**23 (2)**	17 (3)	2 (2)	1 (1)	3 (1)	0 (—)	6 (2)	**6 (1)**
*Exophiala* spp.	**21 (2)**	13 (3)	3 (3)	1 (1)	4 (2)	1 (2)	7 (2)	**8 (2)**
*Purpureocillium* spp.	**22 (2)**	17 (3)	3 (3)	1 (1)	1 (<1)	0 (—)	5 (1)	**5 (1)**
*Cladosporium* spp.	**16 (2)**	13 (3)	1 (1)	0 (—)	2 (1)	1 (2)	2 (1)	**3 (1)**
*Paecilomyces* spp.	**9 (1)**	3 (1)	2 (2)	0 (—)	4 (2)	0 (—)	6 (2)	**6 (1)**
*Geotrichum* spp.	**7 (1)**	4 (1)	0 (—)	2 (1)	1 (<1)	1 (2)	2 (1)	**3 (1)**
*Alternaria* spp.	**4 (<1)**	2 (<1)	1 (1)	0 (—)	1 (<1)	1 (2)	1 (<1)	**2 (<1)**
*Exserohilum* spp.	**4 (<1)**	4 (1)	0 (—)	0 (—)	0 (—)	0 (—)	0 (—)	**0 (—)**
*Verruconis* spp.	**2 (<1)**	1 (<1)	1 (1)	0 (—)	0 (—)	0 (—)	1 (<1)	**1 (<1)**
*Talaromyces* spp.	**2 (<1)**	2 (<1)	0 (—)	0 (—)	0 (—)	0 (—)	0 (—)	**0 (—)**
*Scopulariopsis *spp.	**2 (<1)**	1 (<1)	0 (—)	0 (—)	1 (<1)	0 (—)	1 (<1)	**1 (<1)**
*Cladophialophora* spp.	**1 (<1)**	0 (—)	0 (—)	0 (—)	1 (<1)	0 (—)	1 (<1)	**1 (<1)**
*Lichtheimia* spp.	**1 (<1)**	0 (—)	0 (—)	0 (—)	1 (<1)	1 (2)	0 (—)	**1 (<1)**
*Acremonium* spp.	**1 (<1)**	1 (<1)	0 (—)	0 (—)	0 (—)	0 (—)	0 (—)	**0 (—)**
More than one genus^††^	**23 (2)**	5 (1)	5 (6)	3 (2)	10 (5)	1 (2)	17 (4)	**18 (4)**
Other mold not listed^§§^	**48 (5)**	31 (6)	6 (7)	3 (2)	8 (4)	0 (—)	17 (4)	**17 (4)**

**Abbreviations:** IMD = invasive mold disease; MSG = National Institute of Allergy and Infectious Diseases Mycoses Study Group criteria.

* IMD cases meet a combination of criteria including mycologic evidence (e.g., culture or histopathologic findings), clinical factors (e.g., chest computed tomography findings), host factors (e.g., recent history of neutropenia or ever received a solid organ transplant), and clinician suspicion for IMD as indicated by patient receipt of mold-active antifungal treatment. Potential cases determined not to be IMD cases are included in the surveillance system on the basis of a positive mold culture or a positive *Aspergillus* galactomannan test (serum or bronchoalveolar lavage specimen with index value ≥0.5) but do not meet criteria for an IMD case.

^†^ IMD cases were categorized as MSG-proven, MSG-probable, or surveillance cases on the basis of criteria established by the European Organization for Research and Treatment of Cancer/Invasive Fungal Infections Cooperative Group and MSG consensus definitions for invasive fungal diseases, as revised in 2020 (https://doi.org/10.1093/cid/ciz1008). An MSG-proven case meets laboratory testing criteria of either 1) a positive mold culture from a sterile site or 2) histopathologic evidence of mold angioinvasion of tissue. An MSG-probable case meets clinical and host criteria with at least one clinical factor (e.g., chest computed tomography findings) and one host factor (e.g., recent neutropenia or receipt of a solid organ transplant). Surveillance cases do not meet MSG-proven or MSG-probable criteria but have clinical suspicion for IMD, as indicated by receipt of mold-active antifungal treatment or death within 3 days of specimen collection, potentially precluding receipt of treatment.

^§^ IMD cases with COVID-19 were defined as IMD in patients who also had received a positive SARS-CoV-2 test result documented from 90 days before through DISC.

^¶^ Among IMD patients, non-*fumigatus Aspergillus* spp. included *Aspergillus niger* or *A. niger* species complex (12 patients), *Aspergillus flavus* or *A. flavus* species complex (11), *Aspergillus sydowii* (five), *Aspergillus lentulus* (three), *Aspergillus terreus* (three), *Aspergillus versicolor* (three), *Aspergillus tubingensis* (two), *Aspergillus caelatus* (one), *Aspergillus calidoustus* (one), and *Aspergillus ustus* (one).

** Among these IMD patients, the species of *Aspergillus* was unavailable because the only positive mold test result was an *Aspergillus* galactomannan antigen test (164 patients) or because the species result from the specimen tested was not available (16).

^††^ Among IMD patients with infections involving more than one genus of mold, most frequent combinations included *Aspergillus* spp. and *Fusarium* spp., *Aspergillus* spp. and *Purpureocillium* spp., and *Fusarium* spp. and *Scedosporium* spp.

^§§^ Among IMD patients, other mold taxa identified included *Aureobasidium* spp. (one patient), *Beauveria peruviensis* (one), *Chrysosporium submersum* (one), *Drechslera* spp. (one), *Eutypella scoparia* (one), *Fonsecaea monophora* (one), *Nigrospora sphaerica* (one), *Paraconiothyrium cyclothyrioides* (one), *Parasarocladium* spp. (one), *Phialemonium obovatum* (one), *Pleurostomophora richardsiae* (one), *Pseudallescheria angusta* (one), *Rigidoporus ginkgonis* ([within Basidiomycota], one), *Schizophyllum radiatum* (one), and Zygomycetes spp. (three).

Among patients with any type of IMD, 65% (n = 292) had at least one MSG host factor that predisposes patients to IMD, and 53% (n = 238) had at least one MSG clinical or radiologic factor supporting IMD classification (Table 4). However, 35% of patients with IMD had no MSG host factors identified and would not have been identified as having IMD using classic MSG host risk factors alone. Comparing patients with IMD and without IMD, the presence of certain host factors, including a hematologic malignancy (22% versus 3%; p<0.001), prolonged neutropenia (9% versus <1%; p<0.001), and receipt of a solid organ transplant (27% versus 7%; p<0.001) was significantly associated with IMD. In addition, 59% of IMD cases occurred among patients taking immunosuppressant medication (including high-dose corticosteroids used for >7 days, cytotoxic chemotherapy, transplant immunosuppressive drugs, biologics, and other immunosuppressive medications), a significantly larger proportion than among patients without IMD (19%; p<0.001). Although not frequent among IMD cases, certain conditions were more common among patients with IMD than among those without IMD, including cirrhosis (6% versus 2%; p<0.001), end-stage renal disease (18% versus 5%; p<0.001), and severe burns within the previous 90 days (6% versus 1%; p<0.001). In contrast, chronic obstructive pulmonary disease and asthma were more prevalent among patients without IMD compared with patients with IMD (30% versus 22%; p = 0.003).

**TABLE 4:** Clinical characteristics and risk factors of patients with invasive mold disease — four hospitals, Atlanta, Georgia, 2020–2024*

Characteristic	Potential IMD cases^†^			IMD cases by case type				IMD cases by COVID-19 status^§^		
	IMD case (n = 449)	Not an IMD case (n = 519)	p-value	MSG proven (n = 89)	MSG probable (n = 142)	Surveillance (n = 218)	p-value	With COVID-19 (n = 58)	Without COVID-19 (n = 391)	p-value
	No. (%)	No. (%)		No. (%)	No. (%)	No. (%)		No. (%)	No. (%)	
**MSG-probable case criteria met^¶^**	167 (37)	0 (—)	<0.001	25 (28)	142 (100)	0 (—)	<0.001	17 (29)	150 (38)	0.19
**MSG clinical factor^¶^**	238 (53)	134 (26)	<0.001	41 (46)	142 (100)	55 (25)	<0.001	26 (45)	212 (54)	0.21
Pulmonary aspergillosis	166 (37)	86 (17)	<0.001	11 (12)	111 (78)	44 (20)	<0.001	18 (31)	148 (38)	0.38
Other pulmonary mold disease**	74 (16)	42 (8)	<0.001	7 (8)	33 (23)	34 (16)	0.01	12 (21)	62 (16)	0.35
Tracheobronchial ulceration, nodule, pseudomembrane, plaque, or eschar seen on bronchoscopic analysis	20 (4)	29 (6)	0.46	0 (—)	15 (11)	5 (2)	<0.001	0 (—)	20 (5)	0.09
Sinonasal disease^††^	38 (8)	22 (4)	0.007	19 (21)	14 (10)	5 (2)	<0.001	3 (5)	35 (9)	0.45
Central nervous system infection^§§^	31 (7)	15 (3)	0.004	11 (12)	18 (13)	2 (1)	<0.001	3 (5)	28 (7)	0.78
**MSG host factor^¶^**	292 (65)	93 (18)	<0.001	49 (55)	142 (100)	101 (46)	<0.001	36 (62)	256 (65)	0.66
Neutropenia, prolonged (absolute neutrophil count ≤500/mm^3^ for >10 days ≤30 days before DISC)	39 (9)	1 (<1)	<0.001	10 (11)	22 (15)	7 (3)	<0.001	1 (2)	38 (10)	0.04
Ever received an allogeneic stem cell transplant	18 (4)	0 (—)	<0.001	3 (3)	7 (5)	8 (4)	0.82	2 (3)	16 (4)	1
Ever received a solid organ transplant	120 (27)	35 (7)	<0.001	23 (26)	42 (30)	55 (25)	0.67	18 (31)	102 (26)	0.43
Prolonged use of systemic corticosteroid (>21 days)^¶¶^	69 (15)	32 (6)	<0.001	14 (16)	35 (25)	20 (9)	<0.001	10 (17)	59 (15)	0.70
T-cell or B-cell immunosuppressant medication	223 (50)	59 (11)	<0.001	37 (42)	118 (83)	68 (31)	<0.001	29 (50)	194 (50)	1
Inherited severe immunodeficiency	24 (5)	12 (2)	0.02	7 (8)	10 (7)	7 (3)	0.13	2 (3)	22 (6)	0.76
Graft-versus-host disease grade III or IV (≤90 days before DISC)	1 (<1)	1 (<1)	1	0 (—)	0 (—)	1 (<1)	1	0 (—)	1 (<1)	1
**Hematologic malignancy**	100 (22)	14 (3)	<0.001	20 (22)	54 (38)	26 (12)	<0.001	9 (16)	91 (23)	0.24
Leukemia	51 (11)	4 (1)	<0.001	15 (17)	21 (15)	15 (7)	0.009	7 (12)	44 (11)	0.83
Lymphoma	32 (7)	9 (2)	<0.001	3 (3)	23 (16)	6 (3)	<0.001	1 (2)	31 (8)	0.10
Multiple myeloma	12 (3)	0 (—)	<0.001	3 (3)	6 (4)	3 (1)	0.29	1 (2)	11 (3)	1
Other hematologic malignancy	10 (2)	1 (<1)	0.004	2 (2)	4 (3)	4 (2)	0.92	1 (2)	9 (2)	1
**Solid organ malignancy**	57 (13)	86 (17)	0.10	9 (10)	29 (20)	19 (9)	0.003	2 (3)	55 (14)	0.02
**HIV infection**	19 (4)	18 (3)	0.62	3 (3)	7 (5)	9 (4)	0.88	1 (2)	18 (5)	0.49
Advanced HIV disease***	11 (2)	11 (2)	0.83	2 (2)	2 (1)	7 (3)	0.62	0 (—)	11 (3)	0.37
**Neutropenia (absolute neutrophil count ≤500/mm^3^) documented ≤30 days before DISC**	62 (14)	4 (1)	<0.001	17 (19)	33 (23)	12 (6)	<0.001	4 (7)	58 (15)	0.15
**Lymphopenia (absolute lymphocyte count ≤1,000/mm^3^) documented during 30 days before DISC**	268 (60)	120 (23)	<0.001	43 (48)	102 (72)	123 (56)	<0.001	42 (72)	226 (58)	0.04
**Chronic pulmonary disease^†††^**	183 (41)	242 (47)	0.07	16 (18)	64 (45)	103 (47)	<0.001	17 (29)	166 (42)	0.06
COPD or asthma	98 (22)	158 (30)	0.003	11 (12)	41 (29)	46 (21)	0.01	8 (14)	90 (23)	0.13
Chronic pulmonary disease besides COPD or asthma	102 (23)	130 (25)	0.41	5 (6)	26 (18)	71 (33)	<0.001	10 (17)	92 (24)	0.32
Chronic pulmonary infection^§§§^	19 (4)	26 (5)	0.65	1 (1)	6 (4)	12 (6)	0.23	1 (2)	18 (5)	0.49
**Diabetes**	120 (27)	127 (24)	0.46	28 (31)	42 (30)	50 (23)	0.21	22 (38)	98 (25)	0.06
**Cirrhosis**	29 (6)	11 (2)	<0.001	10 (11)	6 (4)	13 (6)	0.12	4 (7)	25 (6)	0.78
**End-stage renal disease**	81 (18)	24 (5)	<0.001	16 (18)	28 (20)	37 (17)	0.81	15 (26)	66 (17)	0.10
**Severe burn within previous 90 days before DISC**	28 (6)	7 (1)	<0.001	4 (4)	0 (—)	24 (11)	<0.001	1 (2)	27 (7)	0.16
**Immunosuppressive drug use (prolonged, high-dose corticosteroids, chemotherapy, transplant drugs, or others)**	266 (59)	97 (19)	<0.001	41 (46)	129 (91)	96 (44)	<0.001	41 (71)	225 (58)	0.06
Prolonged, high-dose corticosteroid (>7 days)^¶¶^	121 (27)	59 (11)	<0.001	19 (21)	52 (37)	50 (23)	0.006	25 (43)	96 (25)	0.004
Cytotoxic chemotherapy	75 (17)	10 (2)	<0.001	12 (13)	45 (32)	18 (8)	<0.001	6 (10)	69 (18)	0.19
Transplant immunosuppressive drug	130 (29)	35 (7)	<0.001	23 (26)	62 (44)	45 (21)	<0.001	19 (33)	111 (28)	0.54
Other immunosuppressive drug, including biologics and targeted or designer small molecule drugs	74 (16)	22 (4)	<0.001	11 (12)	41 (29)	22 (10)	<0.001	8 (14)	66 (17)	0.71
**Any positive respiratory viral infection test ≤90 days before or after DISC^¶¶¶^**	119 (27)	72 (14)	<0.001	15 (17)	41 (29)	63 (29)	0.07	58 (100)	61 (16)	<0.001
Positive SARS-CoV-2 test result ≤90 days before or after DISC	66 (15)	49 (9)	0.01	7 (8)	17 (12)	42 (19)	0.02	58 (100)	8 (2)	<0.001
Positive influenza test result ≤90 days before or after DISC	9 (2)	5 (1)	0.19	1 (1)	5 (4)	3 (1)	0.42	1 (2)	8 (2)	1

**Abbreviations:** COPD = chronic obstructive pulmonary disease; DISC = date of incident mold specimen collection; IMD = invasive mold disease; MSG = National Institute of Allergy and Infectious Diseases Mycoses Study Group criteria.

* Data are shown as no. (column %). p-values were calculated using Fisher’s exact test.

^†^ IMD cases meet a combination of criteria including mycologic evidence (e.g., culture or histopathologic findings), clinical factors (e.g., chest computed tomography findings), host factors (e.g., recent history of neutropenia or ever having received a solid organ transplant), and clinician suspicion for IMD as indicated by patient receipt of mold-active antifungal treatment. Potential cases determined not to be IMD cases are included in the surveillance system on the basis of a positive mold culture or a positive *Aspergillus* galactomannan test (serum or bronchoalveolar lavage specimen with index value ≥0.5) but do not meet criteria for an IMD case.

^§^ IMD cases with COVID-19 were defined as IMD in patients who also had received a positive SARS-CoV-2 test result documented from 90 days before through DISC.

^¶^ IMD cases were categorized as MSG-proven, MSG-probable, or surveillance cases on the basis of criteria established by the European Organization for Research and Treatment of Cancer/Invasive Fungal Infections Cooperative Group and MSG consensus definitions for invasive fungal diseases, as revised in 2020 (https://doi.org/10.1093/cid/ciz1008). An MSG-proven case meets laboratory testing criteria of either 1) a positive mold culture from a sterile site or 2) histopathologic evidence of mold angioinvasion of tissue. An MSG-probable case meets clinical and host criteria with at least one clinical factor (e.g., chest computed tomography findings) and one host factor (e.g., recent neutropenia or receipt of a solid organ transplant). Surveillance cases do not meet MSG-proven or MSG-probable criteria but have clinical suspicion for IMD, as indicated by receipt of mold-active antifungal treatment or death within 3 days of specimen collection, potentially precluding receipt of treatment.

** If a patient had both *Aspergillus* and non-*Aspergillus* pulmonary mold disease, they would be assigned to the other pulmonary mold diseases category and not the pulmonary aspergillosis category.

^††^ Signs, symptoms, or abnormalities of the sinus, nose, or face or radiologic abnormality of the sinus, nose, or face as observed in computed tomography or magnetic resonance imaging findings.

^§§^ Radiologic abnormality on computed tomography or magnetic resonance imaging findings of head and neck indicating a lesion, mass, nodule, or meningitis.

^¶¶^ Duration with ≥20-mg dose of prednisone equivalent (continuous or discontinuous) during the 60 days before DISC.

*** HIV infection with AIDS (CD4 count <200 cells per milliliter).

^†††^ Chronic pulmonary disease besides COPD or asthma includes *Mycobacterium avium* complex, pulmonary sarcoidosis, bronchiectasis, pulmonary fibrosis, interstitial lung disease, and other disease.

^§§§^ Chronic pulmonary infection includes pulmonary tuberculosis and nontuberculous *Mycobacterium* infection.

^¶¶¶^ Includes SARS-CoV-2, influenza, and other respiratory viruses (i.e., cytomegalovirus, rhinovirus, and parainfluenza). Specifically includes positive tests in the 90 days before or after DISC, to capture respiratory viral infection preceding, concurring with, and following IMD infection or colonization.

Most MSG host and MSG clinical factors, as well as use of immunosuppressive drugs, were observed at a higher rate for MSG-probable IMD than among MSG-proven IMD or surveillance cases, consistent with the case definition criteria. IMD case type did not differ significantly across allogeneic stem cell and solid organ transplant recipients. Severe burns, not considered MSG host factors, were more common among surveillance cases (11%) compared with MSG-proven (4%) and MSG-probable cases (no cases) (p<0.001). Laboratory-confirmed SARS-CoV-2 infections also were more frequent among surveillance cases (19%) compared with MSG-proven (8%) and MSG-probable IMD cases (12%) (p = 0.017).

Although most risk factors and underlying conditions were similar among patients with both IMD and COVID-19 compared with patients without COVID-19, higher rates of prolonged use of corticosteroids (>7 days) (43% versus 25%; p = 0.004) were observed among IMD cases and COVID-19. The difference in corticosteroid use was not observed for longer use of corticosteroids (>21 days).

### Antifungal Treatment, Health Care Use, and Patient Outcomes

Among all 449 patients with IMD, 363 (81%) received antifungal treatment effective against molds, compared with seven (1%) of 519 patients without IMD (Table 5). Among IMD cases, the most common antifungal used was isavuconazole (n = 178 [40%]), followed by voriconazole (n = 142 [32%]), and amphotericin B (n = 93 [21%]). Antimold treatment practices were similar among patients with IMD and COVID-19 and those without COVID-19 but differed by case type. Patients with MSG-probable IMD were the least likely to receive antifungals (57%; p<0.001), compared with patients with MSG-proven IMD (92%) and surveillance cases (92%). Eighteen patients, representing 8% of surveillance cases, did not receive antifungal treatment effective against mold and were classified as having a surveillance case based solely on their death occurring within 3 days of specimen collection.

**TABLE 5:** Antifungal treatment, health care use, and outcomes of patients with invasive mold disease — four hospitals, Atlanta, Georgia, 2020–2024*

Characteristic	Potential IMD cases^†^			IMD cases by case type^§^				IMD cases by COVID-19 status^¶^		
	IMD case (n = 449)	Not an IMD case (n = 519)	p-value	MSG proven (n = 89)	MSG probable (n = 142)	Surveillance (n = 218)	p-value	With COVID-19 (n = 58)	Without COVID-19 (n = 391)	p-value
	No. (%)	No. (%)		No. (%)	No. (%)	No. (%)		No. (%)	No. (%)	
**Antifungal treatment**										
Antimold treatment	363 (81)	7 (1)	<0.001	82 (92)	81 (57)	200 (92)	<0.001	46 (79)	317 (81)	0.72
Amphotericin B	93 (21)	0 (—)	<0.001	42 (47)	10 (7)	41 (19)	<0.001	6 (10)	87 (22)	0.04
Echinocandin	82 (18)	0 (—)	<0.001	23 (26)	11 (8)	48 (22)	<0.001	9 (16)	73 (19)	0.72
Isavuconazole	178 (40)	3 (1)	<0.001	38 (43)	47 (33)	93 (43)	0.15	29 (50)	149 (38)	0.09
Itraconazole	10 (2)	1 (<1)	0.004	1 (1)	2 (1)	7 (3)	0.51	2 (3)	8 (2)	0.63
Posaconazole	62 (14)	0 (—)	<0.001	13 (15)	18 (13)	31 (14)	0.91	5 (9)	57 (15)	0.31
Voriconazole	142 (32)	4 (1)	<0.001	37 (42)	23 (16)	82 (38)	<0.001	12 (21)	130 (33)	0.07
Other drug with antimold activity**	9 (2)	0 (—)	<0.001	5 (6)	2 (1)	2 (1)	0.03	0 (—)	9 (2)	0.61
Antimold treatment with more than one antifungal^††^	149 (33)	1 (<1)	<0.001	46 (52)	25 (18)	78 (36)	<0.001	14 (24)	135 (35)	0.14
**Hospital admission**										
Hospitalized on the day of or during the 6 days after DISC	374 (83)	242 (47)	<0.001	78 (88)	119 (84)	177 (81)	0.38	55 (95)	319 (82)	0.008
**ICU admission**										
ICU admission during the 14 days before but not including DISC	192 (43)	92 (18)	<0.001	32 (36)	53 (37)	107 (49)	0.02	38 (66)	154 (39)	<0.001
ICU on the day of or during the 13 days after DISC	225 (50)	102 (20)	<0.001	48 (54)	59 (42)	118 (54)	0.05	39 (67)	186 (48)	0.007
**Mechanical ventilation**										
Invasive mechanical ventilation during the 30 days before and including DISC	224 (50)	152 (29)	<0.001	36 (40)	70 (49)	118 (54)	0.09	33 (57)	191 (49)	0.26
**Death**										
Death within 3 days after DISC	45 (10)	0 (—)	<0.001	5 (6)	11 (8)	29 (13)	0.08	13 (22)	32 (8)	0.003
Death in hospital	134 (30)	32 (6)	<0.001	27 (30)	33 (23)	74 (34)	0.09	34 (59)	100 (26)	<0.001
90-day mortality^§§^	157 (45)	54 (13)	<0.001	31 (42)	49 (49)	77 (44)	0.57	35 (66)	122 (41)	<0.001

**Abbreviations:** DISC = date of incident mold specimen collection; ICU = intensive care unit; IMD = invasive mold disease; MSG = National Institute of Allergy and Infectious Diseases Mycoses Study Group criteria.

* Data are shown as no. (column %). p-values were calculated using Fisher’s exact test.

^†^ IMD cases meet a combination of criteria, including mycologic evidence (e.g., culture or histopathologic findings), clinical factors (e.g., chest computed tomography findings), host factors (e.g., recent history of neutropenia or ever having received a solid organ transplant), and clinician suspicion for IMD as indicated by patient receipt of mold-active antifungal treatment. Potential cases determined not to be IMD cases are included in the surveillance system on the basis of a positive mold culture or a positive *Aspergillus* galactomannan test (serum or bronchoalveolar lavage specimen with index value ≥0.5) but do not meet criteria for an IMD case.

^§^ IMD cases were categorized as MSG-proven, MSG-probable, or surveillance cases on the basis of criteria established by the European Organization for Research and Treatment of Cancer/Invasive Fungal Infections Cooperative Group and Mycoses Study Group (MSG) consensus definitions for invasive fungal diseases, as revised in 2020 (https://doi.org/10.1093/cid/ciz1008). An MSG-proven case meets laboratory testing criteria of either 1) a positive mold culture from a sterile site or 2) histopathologic evidence of mold angioinvasion of tissue. An MSG-probable case meets clinical and host criteria with at least one clinical factor (e.g., chest computed tomography findings) and one host factor (e.g., recent neutropenia or receipt of a solid organ transplant). Surveillance cases do not meet MSG-proven or MSG-probable criteria but have clinical suspicion for IMD, as indicated by receipt of mold-active antifungal treatment or death within 3 days of specimen collection, potentially precluding receipt of treatment.

^¶^ IMD cases with COVID-19 were defined as IMD in patients who also had received a positive SARS-CoV-2 test result documented from 90 days before through DISC.

** Other drugs with antimold activity used to treat patients included terbinafine, ketoconazole, griseofulvin, and ibrexafungerp.

^††^ The most common combination of antimold treatment for IMD cases was voriconazole and isavuconazole.

^§§^ Ninety-day mortality includes all causes of death ≤90 days after DISC. Ninety-day mortality excludes 2024 cases because death registry data to identify deaths that occurred after hospital discharge were not available at time of publication.

Among patients with IMD, 43% were admitted to an ICU during the 2 weeks preceding the date of specimen collection, 50% required intubation and mechanical ventilation, and the 90-day all-cause mortality rate was 45% (n = 157 of 349; cases from 2024 were excluded from mortality rate calculation because of lack of information on post-hospital–discharge deaths). These outcomes occurred at a significantly higher rate among patients with IMD compared with patients without IMD (p<0.001).

Severe disease indicators were also more common among cases with COVID-19 compared with cases without COVID-19, including higher frequency of ICU admission during the 2 weeks preceding the date of specimen collection (n = 38 of 58 [66%] versus n = 154 of 391 [39%]; p<0.001) and increased 90-day all-cause mortality (n = 35 of 53 [66%] versus n = 122 of 296 [41%]; p<0.001). ICU admission during the 2 weeks preceding the date of specimen collection was more common for surveillance cases (49%; p = 0.023) than MSG-proven cases (36%) and MSG-probable cases (37%). Other outcomes did not differ significantly by case type.

## Discussion

This report summarizes demographic and clinical characteristics, risk factors, health care use, and outcomes associated with 449 IMD cases identified through an active mold surveillance system across four hospitals and associated outpatient clinics in metropolitan Atlanta, Georgia, during 2020–2024. Among cases involving hospitalization, the yearly incidence at the community hospital was 2.8 inpatient cases per 100 inpatient beds; at academic facilities, the pooled yearly incidence was 4.8 inpatient cases per 100 inpatient beds. Although comparable incidence data are lacking, the findings from this report could serve as benchmark data for future IMD surveillance in health care settings, which rely on establishing baseline IMD rates to differentiate between outbreaks and sporadic cases (*9*). Given the low incidence but high severity of IMDs, even small increases in case counts should prompt consideration of a potential health care–associated outbreak. 

Consistent with 2017–2019 pilot data, most IMD infections were pulmonary (68%), and aspergillosis was the most common diagnosis (*8*), accounting for 71% of all cases of IMD from any site. Among the 29% of IMD cases involving other mold infections, fusariosis, mucormycosis, and scedosporiosis were the most common. Data on demographic characteristics were similar to those observed in the pilot study, with most cases occurring among males (65%) and persons aged 45–64 years (43%) or aged ≥65 years (39%) (*8*). These data align with data published in previous studies identifying increasing age and male sex as risk factors for severe fungal infections (*8*,*31*). Similar to the 2017–2019 data, a substantial proportion of IMD cases (35%) occurred in patients without MSG host factors predisposing them to IMD, and outcomes for surveillance and MSG cases were similar, highlighting the importance of considering IMDs among patients without classic host risk factors in public health and hospital-based IMD surveillance efforts to help ensure that clinically relevant cases are not overlooked (*8*,*12*). 

IMD cases were associated with a 90-day all-cause mortality rate of 45%, higher than the rate reported during 2017–2019 (32.7%) (*8*). This higher mortality rate was influenced by the inclusion of IMD patients with COVID-19; these patients experienced more severe outcomes, including higher rates of ICU admission (66% versus 39%) and higher 90-day all-cause mortality (66% versus 41%). However, the 90-day mortality among IMD patients without COVID-19 (41%) was higher than reported before the COVID-19 pandemic during 2017–2019 (32.7%). This observation might reflect the inclusion of patients outside the metropolitan Atlanta area in the data, who would have been excluded from pilot surveillance during 2017–2019. Patients traveling long distances to receive care at these academic hospitals might be more likely to have more complex medical conditions and, consequently, higher mortality rates. When considering only IMD patients from the metropolitan Atlanta area without COVID-19, the 90-day mortality rate (38%) was more similar to, yet still higher than, the 2017–2019 reported rate.

Most of the IMD cases with COVID-19 involved *Aspergillus* spp., with similar frequencies of mucormycosis among IMD cases with and without COVID-19. This finding contrasts with existing studies from the early phases of the COVID-19 pandemic, which reported higher rates of mucormycosis among COVID-19 patients, particularly in India (*32*–*34*). The low frequency of mucormycosis cases with COVID-19 within the EIP surveillance system might reflect the small number of cases in this report or geographic differences in risk factors or the prevalence of environmental molds (*35*).

Of the 968 potential cases reviewed, 46% were classified as IMD cases. This finding underscores the challenges associated with surveilling IMD, because the organizations implementing surveillance might invest substantial time and effort in reviewing a large number of positive mold test findings, many of which could indicate colonization or contamination rather than true infection (*9*,*24*). Most patients with IMD (81%) received antifungal treatment effective against molds compared with a small proportion of patients without IMD (1%), supporting the use of a less stringent surveillance case definition that incorporates clinician diagnosis and treatment to include a broader spectrum of IMDs beyond MSG-defined cases.

## Limitations

The findings in this report are subject to several limitations. First, data were reported only from selected Atlanta metropolitan area facilities, which limits generalizability. Second, the surveillance system might miss potential IMD cases because of the limited sensitivity of mold detection techniques. Third, screening of free text from histopathology findings and DNA-based detection methods such as polymerase chain reaction, metagenomic next-generation sequencing, and other similar assays were not used to ascertain potential cases. However, DNA-based detection techniques have been integrated into the surveillance system beginning with 2025 data. Fourth, the surveillance system does not include a children’s hospital, limiting the system’s ability to include pediatric IMDs. Fifth, the surveillance system did not capture clinical data on patients with cystic fibrosis, limiting the ability to assess IMD status and clinical characteristics in this subgroup. Sixth, the analysis focused on each patient’s initial case of IMD and did not account for subsequent infections. Future analyses should consider characteristics among patients who had more than one IMD. Finally, IMD surveillance relies on medical chart abstraction, which might contain inaccuracies or inconsistencies in the documentation of certain data elements and risk factors.

## Conclusion

This report reaffirms that IMDs are severe infections associated with frequent ICU admission and high case-fatality rates. More than one third of IMD cases identified occurred in patients without classic MSG host risk factors. Although MSG definitions are designed for research settings and prioritize specificity, these findings underscore the need for clinicians and surveillance efforts to consider the possibility of IMDs among patients without characteristic risk factors. To help improve patient outcomes, future efforts could prioritize improving case detection through the development and implementation of rapid, sensitive diagnostic tests for earlier disease identification. Finally, high mortality in the setting of high rates of treatment suggests that new antifungal agents and the optimization of treatment protocols might improve IMD management.

## Footnotes

* 45 C.F.R. part 46.102(l)(2), 21 C.F.R. part 56; 42 U.S.C. Sect. 241(d); 5 U.S.C. Sect. 552a; 44 U.S.C. Sect. 3501 et seq.
